# Book Reviews

**Published:** 1820-01

**Authors:** 


### CRITICAL ANALYSIS

OF

### RECENT PUBLICATIONS, IN THE DIFFERENT BRANCHES OF MEDICINE AND SURGERY;

### SELECT MEMOIRS, AND HISTORIES OF CASES;

### In the Literature of Foreign Nations.

Πατρίδες ἄρα
Ανδράσιν, ὁ πατραῖς ἄνδρες, ἀγαλλόμεθα.

### *Exposition of the Doctrine of* M. Broussais.

As we were preparing to fulfil our promise to give an exposition of the doctrine of M. Broussais respecting the disorganizations consequent on certain species of inflammation, and on phthisis and some other analogous diseases,* a work appeared, bearing a title which could not fail to engage our particular attention:

*The Lectures of Doctor Broussais, on the Gastric Phlegmasiæ called idiopathic continued Fevers by Authors, and on the acute Cutaneous Phlegmasiæ.* †

M. Broussais has for several years promised to develop more fully his doctrine, especially as it regards some particular diseases, than he has done in his *Examen* and the *Histoire des Phlegmasies Chroniques;* but, while this exists in expectation, two of his pupils dedicate to him his Lectures on some of the most interesting points of it, and, in patronizing the work, he might be considered as recognizing the doctrine as his own. But a perusal of these *Lectures* has led us to be confident that this can only be done in a general manner: there is so much vagueness in many parts of them; so little connected reasoning in the exposition of general principles; and, on many occasions, such imperfect accounts of what is clearly and comprehensively displayed in the writings of M. Broussais. How, then, are we to regard these Lectures? and what use are we to make of them? As the exposition of MM. de Caignou and Quemont of certain parts of the Professor's doctrine, with the imperfections, to say no more, consequent on its being derived from instruction from the chair. We shall, then, place these Lectures by our side, whilst we proceed with our own exposition, and refer to them when they speak in a particular manner on points only treated generally in the *Examen* and *Histoire des Phlegmasies Chroniques:* always, however, taking care to designate what we derive from this source, that it may not be confounded with what we give as

* See *London Medical and Physical Journal*, vol. xli. p. 347.

† *Leçons du Docteur Broussais, sur les Phlegmasies Gastriques, dites Fièvres continues essentielles des Auteurs, et sur les Phlegmasies Cutanées aiguës*, par E. De Caignou, de Mortagne, Docteur en Médecine, &c.; et A. Quemont, Docteur en Médecine, &c. 8vo. pp. 290. à Paris, chez Mequignon-Marois, 1819.

the doctrine of M. Broussais; and, in order that no confusion may arise, we shall place within parentheses all that is taken from MM. de Caignou and Quemont.

The nature of the varieties of organic lesion consequent on the various species of inflammatory action, will first engage our attention; and, as we have already given an account of M. Broussais' pathology of inflammation generally, we may proceed immediately to its particular application, and the history of the phenomena resulting from its existence.

M. Broussais considers that a distinction may be made between inflammation as it affects the red capillary, or sanguiferous, vessels; and that of the white, or lymphatic vessels. The former may be either more or less acute; to all the varieties of the latter he gives the generic term *sub-inflammation*; and he calls *mixed* inflammation the simultaneous irritation of the sanguiferous capillaries, and the exhalant, absorbant, and secretory, vessels. Here M. Broussais has not been happy in the choice of his expressions: if the term *sub-inflammation* can convey any precise idea, it must be that of an inferior degree of inflammation, considered absolutely; not, as he intends to signify, a relatively inferior degree of exalted action, when compared to excitement of a similar nature having its seat in other parts of the system: it would therefore have been better to have chosen some term merely designative of inflammation of the white vessels, or exhalants.

The various modifications which irritation, resulting from the impression of a stimulant of any kind, will effect on the organs, may be observed in the following states: 1°, in all the tissues, inflammation of the sanguineous vessels, carried to a high degree, determines the transformation of the part into a red mass, more or less compact, and variable in volume, according to the degree of development of which the organ is susceptible; 2°, a less intense degree of irritation, when it does not terminate by *delitescence* or resolution, transforms the cellular and parenchymatous tissues into a substance analogous to that already designated, but less red and less painful; 3°, in proportion as it extends from the epoch of the invasion of the disease, the induration or red carnification becomes more pale: it terminates by assuming a white aspect, which indicates that the non-sanguiferous vessels have contracted the irritation, whilst that of the red capillaries, which gave the impulse to the disease, has subsided; 4°, when the white vessels are irritated without any apparent previous inflammation, the tissues assume, in the first instance, either the aspect above mentioned, or one of those about to be described; 5°, after this modification of the vitality of the white vessels has lasted for a greater or less length of time, we find united in the tumor, especially when the affected part is of very complex organization, a great number of different matters, such as white, grey, or yellow indurations, in masses more or less considerable in size, and more or less analogous to that which has been called schirrous, cancerous, and tuberculous, matter, with calculous or osseous concretions, in the midst of those masses; and at other times they are interspersed in a confused manner with white, yellow, greyish globules, more or less hard and friable; 6°, a period arrives at which these extraneous bodies, modified by the vital actions, become decomposed, and irritate the adjacent parts, and these become themselves the seat of inflammatory excitement, which destroys them and produces ulcers, the progress of which is often attended with considerable ravages.

Let us now proceed to the development and illustration of the foregoing propositions.

*Inflammation of the sanguiferous vessels* may be dissipated at the end of a certain period; and this is termed *delitescence,* or resolution. (Some evacuation or other, termed critical, is then observed, say MM. De Caignou and Quemont: sometimes abundant sweats appear, the cutaneous exhalents then assuming more activity. Sometimes there are copious evacuations of urine, with a copious deposition of a mucous sediment, forming a cloud, in the middle of that fluid. The former phenomenon is observed in phlegmonous and parenchymatous inflammations of considerable extent. The local consequences are a softening and subsidence of the tumefaction of the part, accompanied by absorption of the fluids evacuated during the inflammation ; and often no sensible traces are left in the cellular tissue. These observations apply especially to delitescence. When it may be said to terminate in resolution, there are sometimes found adhesions of the cellules of the tissue above mentioned ; which is also remarked after this mode of termination in serous membranes. In the mucous membranes, the exudation, in the first instance wanting, becomes serous and abundant; then more thick, and of a mucous appearance: it approaches to the nature of pus. The membrane suffers no alteration in its texture. In the parenchymatous structure, the product has a cream-like appearance. The resolution takes place in the same manner as in the mucous membranes. In the cutaneous texture, it terminates by desquamation. In the fibrous textures, it leaves a coating over the parts, that gradually disappears. It is not known how it terminates in the synovial membranes. In general, when not evacuated externally, the product is absorbed, and then thrown out of the system by some of the excretory organs.)

2°. It may produce a too-rapid sanguineous engorgement, which overpowers the vitality of the part: this is gangrene by excess of inflammation. (In the lungs, local death is manifested by a real apoplexy affecting the organs, so that their functions are suddenly suspended by the afflux of blood and the compression of the bronchial vessels, produced by this fluid. In this case, general death takes place sooner than gangrene, from the importance of those organs. This phenomenon prevents our finding, on opening the dead body, any trace of putrifaction. Gangrene of the lungs is extremely rare: it sometimes takes place, especially as a consequence of deleterious causes ;* and then only one lung can be affected, the sound one supplying the functions of the other, and prolonging life sufficiently to permit it to putrify. We have observed a few cases of this kind.)

* The expression of MM. De Caignou and Quemont:—perhaps inspiration of some gaseous fluid is meant.

3°. It may disappear, because of the organs dying from previous debility of the vessels, or from the influence of a deleterious or sedative cause, which may itself have been local irritation: this is gangrene by debility. (Gangrene from a deleterious cause, malignant pustules, and carbuncles, should be arranged with those which depend on debility, because the poison destroys the vital powers in the midst of the inflammation.)

4°. By producing suppuration; which supposes that the sanguiferous capillaries terminate on a cellular or serous tissue capable of expansion. (In this case, the irritation is not confined to the red capillaries; and the proofs of it are, (*a*) that inflammation may persist in them for a long time without producing pus; (*b*) that pus may be formed by cellular and serous tissues, without having been preceded by inflammatory symptoms. The termination of inflammation that produces pus, supposes two things: (*α*) that the part is furnished with an expansible cellular and serous tissue; (*β*) that the inflammation is communicated to this tissue in the peculiar degree which produces suppuration. The cellular tissue existing in very small proportion in the dura mater, true pus is very rare. The secreted liquid is rather transparent and gelatinous: collections of true pus are, however, found there. The product of inflammation of the *arachnoid membrane** resembles that of the pleura and the peritoneum. Irritation of the sanguiferous vessels may exist without the formation of pus. In setons, suppuration is often suddenly suppressed, although the irritation and redness continue to exist. This phenomenon may be observed in all the tissues susceptible of inflammation. We may then conclude, that suppuration is only an incident in inflammation. If we see phlegmasiæ become chronic because they are kept up by the pus, there are also others found existing a long time without this cause. There are examples of persons who, for several years, have presented all the symptoms of inflammation of the viscera, in whom, on examining the dead body, not the slightest trace of pus is present, nor even the smallest appearance of suppuration.)

5°. *It may continue in a chronic character:* its effects then present a multitude of varieties, according to the texture of the part, its vitality, and the general disposition of the patient. The principal results are: (*a*) sometimes it is confined to the sanguiferous capillaries, permanent congestion in which presents the red induration, which takes different names according to the organ in which it is seated: in the skin and cellular tissue, callosity; in the mucous membranes, no particular name; in the lungs, hepatization, &c. But all the tissues do not submit themselves to this form of alteration. If the chronic inflammation

* *Meningette* is the term used by the writers of the Lectures:—we think they mean the *arachnoid* rather than the *pia-mater*. *Meningine* we translated *dura-mater*. Our translation appears to be correct only from the pathological observations applied to those terms. M. Broussais has never used them in his writings; and, if he has orally, it is but lately that he has done so. The writers of the Lectures should have explained what they meant. This must be placed amongst the multitude of faults in their publication of the Lectures of the Professor at the Val-de-Grace.

is not confined to the red vessels, then, (*b*) in the cellular tissue, it will produce pus, which, if it be retained and decomposed, may itself keep up the inflammation, until the general exhaustion of the vital powers, is the consequence; (*c*) in the membranes, the results will be different according to the tissues which exist in them, and the disposition of each of them to contract irritation: it may then develop phlyctenæ, herpes, dartres, indurations, ulcers, pustules, horns, tubercles, &c.: in the mucous membranes, a copious secretion of mucus, more or less resembling the pus of phlegmon; a concrete, cream-like, suppuration; and the ulceration of cryptæ: in the serous membranes, an exudation of a multitude of varieties of character; (*d*) in the parenchymatous secreting organs, suppression, augmentation, or alteration, of the product of their secretion; (*e*) in all the organs, without exception, it may develop the cellular tissue, produce fasciæ of various kinds, and alter the lymphatics, so as to present all the degenerescences termed cancer, phthisis, scrofula, and fungus hæmatodes.

The alterations of structure arising from *inflammation of the white vessels*, appear under various forms, according as different species of vessels are particularly affected. (1°. The tuberculous. The glands which present this alteration, first acquire increased size and hardness; from red they pass to dull white, then become soft, and take the consistence and colour of cream. It is seen in the glands of the bronchiæ and mesentery. The same alteration is observed in the absorbent vessels of the cellular and serous tissues. 2°. The lardaceous. It is so termed from the resemblance it has with lard. Its seat is in the cellular tissue, which it renders, as it were, infiltrated with gelatine. It is not possible to say what are the vessels which most contribute to its formation. 3°. The encephaloid or cerebriform. This is white and softish, and is observed in the cellular tissue. It nearly approaches to the tuberculous. 4°. The melanose, thus termed because of its blackish colour, does not make a distinct species from the preceding. The black colour affects indifferently all the degenerations. 5°. The cartilaginous, the osseous, and the calcary. The two former are sometimes organized, and are met with in the ossification of arteries and of the cellular tissue. The third is inorganic: it is found in the midst of the depositions of matter established in the different disorganized tissues. It is also observed in some glands. 6°. The degeneration into erectile tissue, thus termed from its analogy to that of the corpora cavernosa. It appears in the skin, in nævi materni and fungus hæmatodes. The nature of it is not well known, but it appears to appertain to the sanguiferous capillaries. It has been seen in the different viscera. 7°. The polypous, or fungous degeneration. It is a vegetation which arises from the cellular tissue. This vicious nutrition is sometimes observed with the alterations already spoken of. It contributes, perhaps, to the carcinomatous and cancerous degenerescence, which forms the highest point, and, as it were, the termination, of the disorganizations. 8°. Encysted degenerescences, and the transformation of one tissue into another, without speaking of ossifications and accidental formation of cartilage. It is thus are formed cysts endowed with hair, species of accidental murous membranes, and that certain cysts are developed around coagula of blood, and foreign bodies present in their interior a smooth exhalant surface, analogous to that of the serous membranes.)

The many points in which M. Broussais, Dr. Baron, and Mr. Langstaff, concur on this subject, will be immediately discerned. The difference between them may probably depend more in words than in ideas. In using the term inflammation, M. Broussais only means to signify a certain alteration in the action of the vessels, which he believes to be greater than the natural action, because it arises from the impression of stimulants, and it passes to what is evidently inflammation, and returns again to the former state, merely from the impression in a greater or less degree of the same causes, and with only a variation of the same general phenomena. Somewhat seems to depend, too, on the different constitution of different individuals; for we see that, from identical causes, sanguine robust subjects are affected with violent phlegmasiæ; whilst the less strong, slender, lymphatic, feebly developed, subjects, have them in a less pronounced degree, and of the chronic character from the commencement. It is in the latter that irritation is tacitly communicated to the lymphatic vessels, and insensibly leads to tuberculous and other disorganization. We find also, 1°, that, whenever those disorganizations have been preceded by general irritation of the sanguiferous system, their progress is more rapid in proportion as that has been more violent. 2°. When they have not been preceded by such irritation, the cause has nevertheless always been stimulant, as is proved by their etiology; and their progress may be accelerated or retarded, by calming or rendering more powerful the action of those stimulant agents. 3°. They are always seen developed in those tissues endowed with the greatest vitality, in the periods of life when those tissues enjoy the greatest activity, and never in parts which have become affected with paralysis. M. Broussais shows, too, that there is no tissue in which the disorganizative irritation may not be developed in two different manners: 1°, with violent re-action of the sanguiferous system; 2°, without being preceded by this re-action. It is important to remark, that these affections influence other parts, according to the common laws of sympathy: as this will explain the transmission of the disease from one part to another, &c. and all the circumstances supposed to indicate the existence of a *specific virus.*

We have not terminated the exposition of the doctrine of M. Broussais: something should be said respecting ulceration in those degenerescences. Whenever the phenomena which accompany cancerous decomposition, or the ulceration of schirrous and tuberculous masses, are observed, we find, says M. Broussais, that inflammation of the sanguiferous vessels is developed, and that the progress of the disorganization thence arising is more rapid as that inflammation has been more intense. We always see simultaneous inflammation of the sanguiferous and non-sanguiferous vessels, for that constitutes the character of disorganizing and propagative inflammation; and this mixed inflammation is never developed but under the influence of either local or general excitant causes. All those substances, then, which stimulate locally, or which sympathetically exalt the actions of the nervous and sanguiferous systems accelerate the progress of the disease.

We have not yet spoken of the influence of the local diseases above designated on the functions of other parts of the system; but, we shall now give a general summary of the phenomena which thence result.

Inflammation has a more powerful influence on the exercise of the functions in proportion to the greater degree of its energy, and *vice versâ*. Thus,

1°. From *inflammation of the sanguiferous vessels of the phlegmonous character*, we observe fever, more or less general uneasiness, very severe disorder of the nervous functions, and derangement of the secretions; and, from the *progress of the disease and its prolongation into the chronic state, with suppuration, ulceration*, &c. severe hectic fever, consumption, and marasmus.

2°. From *inflammation of the sanguiferous vessels of organs but little supplied with red capillaries, or extended in membranes*, less acute fever, considerable nervous disorder, and corresponding derangement in the secretions; but all these accidents are not constant, and often some of them are present in but a very slight degree. From *its prolongation into the chronic state*, with suppuration, ulceration, &c. mild hectic fever, often hardly decisively marked; slow consumption, and marasmus proceeding but slowly; unless the inflammation occupies the organ which presides over assimilation: in this case, the extenuation is prompt, considerable, and does not depend on the fever. Dropsy may occur, especially if the hectic is but slight in degree.

3°. From *inflammation of the lymphatics*, or irritation of the white capillary vessels, no fever, no sympathetic disorder; unless when complicated with the preceding species of irritation. From *its long duration*, if the irritation of the white vessels is pure and simple, alteration of nutrition, derangement of the serous and lymphatic secretions, and dropsy. From *ulceration of the degenerescences*, if combined with a high degree of phlogosis of the sanguiferous vessels, very rapid hectic fever, and very considerable marasmus: if combined with less degree of such inflammation, the same phenomena, only less intense and less rapid in their progress and succession.

Before we proceed to M. Broussais' doctrine of other morbid affections, we shall adduce some remarks on the treatment of some of those that have already engaged our attention. It is to the organic degenerescences that we shall confine our remarks; as, apparently in a great measure from the notions that have been entertained respecting the origin of the greater part of them, they have been generally considered as incurable. Supposing them to be formed of new tissues spontaneously developed, without any previous inflammation appearing to give them birth, it has been concluded that they are parasitic animals, as their structure also differs from the natural membranes, the appearance and increase of which have been considered equally inexplicable, and against which all the efforts of art have been supposed to be unavailing. M. Broussais vigorously attacks this despairing doctrine. We retrace our steps to adduce in detail some of his remarks on this point, which we only gave in a general manner in the foregoing exposition. After having collected and combatted the arguments of his adversaries, he establishes, "that the non-sanguiferous vessels of different orders, whether exhalents, absorbents, or secretors which are endowed, as Bichât demonstrated, with a peculiar irritability and species of vitality necessary for the exercise of their functions, are susceptible of aberrations in their properties, independently of that exaltation of action of the sanguiferous system which we qualify with the term inflammation; nevertheless that, in the most ordinary cases, this aberration is communicated to them by the inflammatory state, as all those know who have taken the trouble to trace, in a great number of instances, the origin of congestions, schirrosities, &c. This fact leads us, then, to think that, in the cases where the tumefactions are not preceded by the inflammatory state, the aberration which disorganizes them is not less the effect of an exaltation of their organic action. The causes which preside over their formation in the latter circumstances, show the propriety of this manner of regarding them; since these causes may be always reduced to either an immediate or a sympathetic excitation, which has augmented the vital action in the degenerated part at the expense of that of others. I may here repeat what I said in speaking of hæmorrhagies and inflammations; which is, that these alterations, or degenerations which are developed in the non-sanguiferous vessels, are ordinarily manifested in the organs endowed with the greatest vitality: in those where the vessels in question are animated by numerous sanguiferous capillaries; in those where we find, with those conditions, a great number of nerves, much sensibility, and a close texture, which opposes itself to a free development of the phenomena of inflammation;—such are the breast, the facial region, the most firmly bound and most sensible parts of the digestive canal, the neck of the uterus, the parenchyme of the lungs, and, lastly, all parts of the body, (for the same tissues every-where exist,) when a violent cause of any kind has for a long time exalted their vital properties."—*Examen*, pp. 298-300.

M. Broussais has on these principles endeavoured to prove that it is possible, if not to obtain the resolution of the tumors formed by those new tissues, at least, to prevent their formation in the greater number of cases. If, indeed, they are ordinarily the result of prolonged irritation, it is evident that, in combatting this irritation by appropriate means, we may evidently prevent the degenerescences that arise from it in the greater number of instances. When the disease is seated in external parts, emollient applications, local blood-letting, and a severely-restricted diet, are the means to be employed in their cure. The body should be made to live at its own expense, in a manner, for a certain length of time. It was by following the latter indication that Girardot had such astonishing success in the treatment of scrofula. *Fames rectè ordinata, scilicet nimia et prava ciborum ingestio cautè et indesinenter prohibita*, he regards as the chief means of cure; and, he continues, *frustrà sudaverit medicus strumosam pharmacis insequendo diathesim, nisi congururenter famem foverit.* Sometimes, when inflammation of the sanguiferous vessels is not conjoined, stimulants may be employed to change the mode of irritation locally, or develop the action of sympathizing organs, and thus produce revulsion. Sometimes specific stimulants are required, as that of mercury in syphilis, &c. It is said in the Lectures, that M. Broussais does not believe that any of the medicines employed with this view go directly to the organs they seem especially to affect: the latter are acted on solely by their sympathetic connection with the stomach, which produces different effects on different parts, according to their peculiar sympathetic relations to it:—Exactly the opinions of Dr. CHAPMAN, of Philadelphia.

It now remains for us to show how far the foregoing principles will explain the phenomena of some organic diseases, which, when seated in certain organs, have received peculiar names,—as phthisis, scrofula, &c.; we shall also give a general account of M. Broussais's theory of scurvy; and then we shall attend more particularly to the *Lectures*, for the purpose of illustrating some of the principles developed on former occasions respecting gastric inflammations.

---

WHILST waiting for the completion of the sources whence the means for giving a full exposition of the medical doctrine now prevalent in Italy must be derived, we received some fasciculi of a work published at Bologna, for the express purpose of tracing the origin and progress of the most important parts of that doctrine,—those which relate to febrile diseases, and the mode of agency of certain medicinal substances on the human body. These fasciculi contain analyses of the Treatises of RASORI and GUANI; and, since we have not yet received those works, and that a full account of them, as containing the bases of the modern opinions, must form a proper pröemium to a general summary, we shall now give a translation of those analyses; which we do with full confidence that they are effected with accuracy, from our knowledge of the talents of the author: but, as he has chosen to remain *sub umbrâ*, we cannot show our readers on what grounds that assertion is made. At present we adduce no reflections of our own on the sentiments about to be laid before the reader; we only premise that, although we adopt the following articles, we do not identify our opinions, on all occasions, with those which they contain.

*The History of the Petechial Fever of Genoa in the Years* 1799 *and* 1800; *and some Hints respecting the Origin of Petechial Diseases, &c.* By G. RASORI, Doctor in Medicine of the first Class, &c. Milan, 1813.*

At the commencement of the present century, the opinions of JOHN BROWN were adopted by the greater number of physicians in Italy; and that scholastic chief, not less celebrated in Germany than in the former nation, acquired from day to day additional reputation and

* *Storia della Febbre Petecchiale di Genova, negli anni* 1799 *e* 1800, *ed alcuni cenni sull' Origine della Petecchiale. Terza edizione, aggiuntavi un' indagine intorno ai comuni Errori d'Osservazione nella Terapeutica di questa Febbre*, Di G. RAZORI, *Protomedico, ec.* Milano, 1813.

credit. The opposition of the followers of the more ancient doctrines became gradually less active; and many of those antagonists, amongst whom were observant physicians who, finding that some of the most famous precepts of Brown were deleterious to mankind, and contrary to their own experience and that of their predecessors, whilst they followed in their practice the instructions derived from the latter sources, did not venture to censure them publicly, so great was the fanaticism with which the Brunonian theory was almost universally received. At this arduous period of enthusiasm appeared the celebrated work of Giovanni Rasori on the epidemic of Genoa in the year 1800; and the unbounded reputation of the Scotch physician immediately became somewhat shaken, and his maxims regarded in Italy with less idolatrous veneration.

In 1799 there appeared in Genoa, affecting simultaneously many individuals, a fever, which, by the most evident signs, was discerned to be petechial. It made more progress towards the middle of the year 1800; and it seemed to be from Nice that its origin was derived. It propagated itself, as usual, principally, and with most severity, when the patients were most accumulated; but, on some occasions, it could not be discerned by what secret way the disease had been communicated to persons the most cautious and retired, who had not escaped its attack. Those circumstances, however, present nothing that is singular, or not common to other epidemics. Neither were there any extraordinary facts of importance in the progress of the morbid phenomena, which are not unfrequently remarkably altered and modified in epidemic maladies.

This disease commenced with heavy pain in the head, or with a sense of vacuity; sometimes to this there was added a slight degree of mental derangement, which afterwards became furious delirium. Preceded by alternations of shivering and heat, or by heat alone, or by neither, the fever at length manifested itself, simulating, in some cases, the access of a mild catarrhal affection. Almost all the patients complained, from the first period of the disease, (which it is important to remark,) of remarkable muscular debility, which in some was so great, that they fainted, on making the slightest exertions. The other phenomena of the first stage were in no respects novel. There were often pains in the joints, universally or locally, and especially in those of the extremities. The countenance varied in its appearance: it was in some instances hot and tumid, with the eye lids a little closed; in others pallid, but never of the intense white colour, and singular depression of physiognomy, that accompany fever really nervous. The eyes, in general, had an appearance of vivacity, and were more lucid than ordinary. The skin was very warm, but not of a burning heat. The thirst not very excessive. The tongue often, in the beginning, of the natural appearance; in the course of the disease, under the continued operation of purgatives, covered with a white, and, in some cases, with an intense yellow, crust. Sometimes humming and singing in the ears on the first days, were the precursors of deafness, which, appearing towards the latter stage of the disease, announced approaching dissolution. Obstinate watching and inquietude, increasing, and then changin into stupor, under the inconsiderate use of opiates. The pulse, at the commencement, from 80 to 100 in a minute; *small, obscure;* rarely strong and full, and sometimes singularly weak and indiscernible. The urine was too various to permit any certain signs to be deduced from its appearances. Copious sweating, in many cases, during the first few days, and especially in the night. The bowels were constipated, but particularly susceptible of the influence of purgatives, and subject to diarrhœa. Epistaxis was not unfrequent, and was always beneficial in proportion to its abundance.

Matters went on thus for three, four, or five, days. The disease then became aggravated, and accompanied with convulsive movements. Fainting, sometimes without evident cause, or in the act of evacuating the bowels; and commonly subsultus of the tendons, and tremulous tongue. Deglutition was not always perfectly free. The pulse varied in diverse individuals, and its rate was different at different times of the day. Generally, or most frequently, it was intermittent, small, or so sunk as to be totally lost; but it ordinarily became strong when the disease was more advanced, and under an energetic depletory mode of treatment. Petechiæ, or an analogous eruption; miliary spots; one or both then appeared; but they were not present in the mildest cases of disease, and they were copious in proportion to its severity. In one case, an erysipelatous affection occurred simultaneously in the head and face. In another, the skin and albuginea were of an intense yellow colour. Sometimes there were exanthemata, which left the skin rough and scaly. In almost all cases there was delirium or stupor, or alternation of both. The former was so ferocious in some cases, as to require the patient's liberty of movement to be confined to prevent suicide, to which there was inclination. They then refused to swallow; and the tongue became swelled, dry, and of a deep-red or black colour, as well as the teeth. Meteorism was not rare; occurring, for the most part, in those who lived after suffering abundant evacuations by stool, which were sometimes sanguineous and profitable. Ischuria was protracted, in one case, however, to the period of convalescence. Hiccough was very obstinate. Vomiting was rare, which also generally, when it happened, arose from large doses of tartrite of antimony, and was attended with pain and difficulty. Respiration was but very rarely affected. Restlessness arose almost solely from the treatment with stimulants. In one instance only, the disease commenced with all the symptoms of peripneumonia. Convalescence presented nothing worthy of particular notice. It was accompanied with, or ordinarily announced by, frequent spitting; without, however, any affection of the chest being manifest; and, on the decrease of fever, or on the cessation of delirium and stupor, with great sadness, irascibility, or very remarkable fear of death.

We may sufficiently understand, from what has already been said, what species of disease the celebrated Professor had to contend with. How did he cure it? Having long modelled his medical doctrine on the principles of Brownism, he was naturally conducted by the predominant idea of asthenia. Every thing conspired to lead him to form that opinion of its nature. He considered the circumstances which preceded the first breaking-out of the disease, and those which accompanied its attack and progress. Those were, the flow into Genoa, in the end of the summer of 1799, of many Cisalpines and other refugees, victims, generally, of the most severe depressing passions, exhausted by excessive fatigue, long exposed to heavy rains, and confined to very ill and insufficient nutriment. What gave some weight to the indications from the symptoms, were the evident and remarkable prostration of strength from the onset of the malady, the irregularity and smallness of the pulse, and the disposition to fall into syncope. On contemplating the external characters of the disease, the apparent phenomena clearly announced, at least, a typhous or nervous fever, which Brownism referred to the most depressed state of asthenia. On reflecting that the typhus was petechial, he was induced to consult what the reformer of Scotland said on the subject; for though, considering it as the product of a contagion, it would have taken its place in the fourth division of local diseases, yet, from its being complicated with phenomena of universal affection so worthy of consideration, and caused by circumstances so debilitating as those above described, it should manifestly enter into the ordinary class of universal diseases and of the most profound asthenia; and as such he was obliged to regard it. Lastly, judging, as they are, the authors of former ages worthy of being consulted, he wished to know what in this case would be their advice: the doctrines of these, although in general contrary to the maxims of Brown, did not, however, refute him in the notions that, where such sudden langour of the vital actions appeared so evident, the existence of pathological debility was undeniable, and required us to oppose it immediately with all the measures considered to be corroborant.

According to those principles and those reflections, Professor Rasori judged that, whether the asthenia was direct or indirect, (although he had already formed many doubts respecting the propriety of the extensive application accorded to the latter,) it was right to stimulate; but he fortunately fulfilled this precept in a manner somewhat less determinately than the remains of the doctrine of Brownism inculcated. Every thing, excepting subtraction, stimulate, according to Brown: water and a diet of vegetables, which debilitate, debilitate because they stimulate too little. Emetics and purgatives, which debilitate, debilitate because the quantity of the stimulus of both together is less than the quantity of stimulus subtracted by the evacuations they promote. G. Rasori was not well assured of the correctness of those notions; and he could not make up his mind to think all the former physicians had been in error who believed that there were some substances which *directly* debilitate without producing subtraction,—such as *sedatives*, *refrigerants*, *dilutescents*, &c.; or, speaking more rigorously, which directly cause organic actions precisely opposite to those produced by stimulating powers. Many doubts, at least, on those points existing in the mind of the author, a most happy consequence arose: it was, that, intending to stimulate, to free himself from the danger of contradiction in the means of cure, he determined not to excite with those indeterminate remedies whose qualities did not admit of certain decision, but solely with those on whose stimulant qualities there was no ground for controversy.

Having once determined the principle on which he should stimulate his patients, and considered, on the one hand, that this should be conformed to in that prudent manner which is proportionate to the severity of the disease, without exceeding, or falling short, of the proper degree; and, on the other hand, that, by employing only certain and unequivocal stimulants, he should, in case he had adopted an erroneous mode of treatment, immediately detect this by the evident pernicious results; and, if an appropriate one, he might hope that nothing should prevent its general success. Having thus firmly settled his mode of conduct, he used as a vehicle a decoction of China-root, to which were added Hoffmann's anodyne, the liquid laudanum of Sydenham, and wine; an appropriate diet was conjoined, and all watery beverages proscribed: but he soon had to repent of his conduct, and of the Brunonian notions which had till then influenced his mode of contemplating the disease.

Whilst this decisive method was put in practice, at the end of from twenty-four to forty-eight hours, sometimes sooner and sometimes later, matters had manifestly assumed worse appearances. They were not the ill consequences of the disease, which, whatever may be the mode of treatment, will increase, and run through its stages. The malady was too promptly and too manifestly exasperated; without any gradations, any proportion to the preceding state, or to the period that had intervened. The frequency of the pulse was increased, and its hardness more perceptible; the face red; the eyes sparkling; and the respiration less easy. Besides, as the author acutely reflected, diseases really asthenic have not properly that character of obstinacy, by which they will become augmented in spite of an appropriate method of cure continued for several days: this obstinacy alone might lead to a strong presumption of the sthenic nature of the disease. What, then, was the resolution to be taken in such circumstances; and what change was to be made in the mode of cure?

Those who know with what difficulty a change of opinion on any subject takes place in a man who has long formed but one respecting it, will agree with us, that it is difficult to understand how the circumstances above described could produce such a revolution of ideas contrary to doctrines so long entertained, and lead to an opposite mode of treating the disease. A Brunonian, in spite of those occurrences, would have scrupled to renounce in a moment the favourite principles of his master: he would not have believed his eyes; he would have taken refuge in a thousand extraneous circumstances, and supposed that he had not employed appropriate stimulants, or given his remedies in doses sufficiently strong for the intensity of the disease. He would have opposed to his own experience a heap of ancient and modern authorities; and, proceeding with his stimulants, would at last have assumed an air of wonder at the insuperable nature of the malady against which he had to contend. We do not speak of things impossible. In the petechial epidemic, which, from the commencement of the present century, has overrun all Italy, numerous Brunonians must have seen similar phenomena to those observed by Professor Rasori; but we do not find that any change has taken place in their practice, even although the work before us has circulated through the hands of physicians.

The author's merit will be still more evident, when it is considered that, although he in the first instance had recourse to the Brunonian method, yet, knowing the coolness required for correct observation, and the difficulties attendant on it, and how many important things ordinarily escape our attention, he resolved to have recourse to the *experimentum crucis,*—that is, to a directly opposite mode of treatment. He acted with caution in the first instance, but afterwards with more courage; but it was not long before he was convinced of the propriety of this second attempt. As the disease, on its first appearance, presented a character of mildness, and experience had not confirmed the propriety of his conduct, he was timid: his measures then were equivocal or inactive, consisting wholly in a sort of expectation. He abstained from abstraction of blood; and merely moderated the diet, relinquished all heating substances, and directed the free use of diluent drinks. The only at all active medical measures he employed were, acidulated drink in large quantity, tamarinds, nitre, and other neutral salts. When the epidemic, in the height of its fury, showed itself generally with a much more serious aspect, and he had sufficiently perceived with what courage and certitude he might act, he no longer stood on the defensive,—no longer doubted. Although it may be considered that he carried debilitating measures to a small extent, according to the idea of some furious partizans of the depletory method, yet it cannot be said that they were not used in a decisive manner. He bled from the arm once or twice at the commencement, and, in one rare case, even after the tenth day of the disease; and then, if bleeding seemed to be indicated, he applied cups to the shoulders, or leeches to the temples or neck, sometimes so as to obtain, on each occasion, eight or nine ounces of blood. Then he resorted to the use of antimony, in its preparations, as kermes or as tartar-emetic. He prescribed of the latter four, six, eight, and sometimes more, grains, daily, to be taken in a large quantity of some ordinary aqueous drink; and he proceeded thus until the decisive epoch of amendment. Sometimes he used kermes combined with nitre, in the proportion of a scruple of the latter with half a grain or a grain of the former, every two hours. Sometimes he alternated the two remedies. In the mean time he did not neglect the use of purgative glysters, for the most part rendered so by tartar-emetic; copious aqueous and vegetable beverage, chiefly, in those who could easily obtain it, of decoction of tamarinds; free circulation of cool air; the least and most light covering on the bed; vegetable jellies for the rich, and aqueous fruits for the poor; and in all a very restricted quantity of food. Opium, camphor, China-root, wine, alcohol, ether, ammonia, epispastics, and vesicatories, were never employed. What were the results? Of a very great number of patients that came under the author's care, not one died, although the disease was so far from being innocent, that, from April to October, 1800, there was a mortality of 7,813 individuals.

It would, however, be easy to show the severity of many of the cases treated by the author, by transcribing from his work some of the accurate histories with which it is accompanied; but for these we must refer to the original: we shall here only adduce some of the reflections that arise from the consideration of the circumstances above detailed.

It appears, then, that a method rigorously, and even energetically, antiphlogistic, refrigerant, and depressatory of stimulus and excitement, used from the onset of the disease until its termination, and steadily maintained, even when the phenomena indicated, in a manner the least subject to dispute, the presence of the pretended malignant, nervous, and asthenic, period; not only is not injurious, but affords such manifest assistance, as to conduct to health, not a small number of patients, but all of them. Here, then, is a fact absolutely new, at that epoch in the records of our art which, as it were by enchantment, made all those physicians who reason, lower the veil from before their eyes, and overthrew the long-venerated idol of malignity, of the believed immediate progress in typhus to real, to pathological, debility, and of the pretended necessity of using cordials and restoratives in the cases alluded to.

In all past times, commencing with the epoch in which the petechia first appeared, this disease has been cured, and in general is now cured, by the most judicious physicians, by depressing means; and there were many who had to felicitate themselves, before Professor Rasori, on the success of their treatment of that disorder. Even in the existing temple of the fanaticism of Brownism, many Brunonians, moderating the incendiary nature of their method by mingling with their real stimulants other substances, which we shall hereafter prove to be of an opposite class, are less destructive in their practice, and may boast of some success; but, unfortunately, the opinion has been invalidated amongst those physicians that made them see a species, a form, of the disease, a period in the same, or sometimes a particular epidemic character, where the apparent langour of the vital powers, the supposed colliquative phenomena, the diarrhœa, the meteorism, the excessive epistaxis, the tremulus of the muscles, lypothymia, the smallness and lowness of the pulse, the general depression of the cerebral functions, the subdelirium,—announced, more or less clearly, the necessity of a stimulant, heating, analeptic, and cardiac, mode of treatment, the occasion for invigorating by stimulating means, and the precept to abstain rigorously from all that could depress or debilitate. Nevertheless, Pier da Castro had said on the same occasion, "*Detrahendus statim sanguis est per venam sectam, maxime quantum fieri possit ab initio. Est enim hæc operatio tanti momenti, ut illa neglecta, vix faustum eventum in hac febre vobis liceat sperare, quantum cumque blateret indoctum vulgus:*" and he added, "*Extrahite in principio alacriter sanguinem;*" and prescribed largely, at the same time, the use of mild purgatives, cupping with scarification, the application of leeches to various parts, emollient glysters, cold and acidulous drinks, affusion with water and oil mingled with nitre; not hesitating to subjoin, "*Si vero nulla sunt plenitudinis signa, cavete in hac febre, cave*

***pejus et angue, a venæ sectione, post punticularum præsertim exitum;*** and was careful to cease or moderate the use of the measures indicated, as soon as there appeared *colliquationes*, *syncopes*, *exsolutiones*, *anxietates;* and directed, what is more, on such occasions, food sometimes of roasted meat, with cordial drugs,—as amber, musk, generous wine; and recommended many alexipharmacs, amongst which were, as there might appear occasion, theriaca, mithridate, diascordium, and some compound remedies, with opium, musk, and ambergris. Not only did Pier da Castro adopt this method, but, ascending to still earlier authors, we find the same cautions, the same indications, as far upwards as the time of Borsieri; and so enracinated in physicians were these documents and prejudices, that, even after the third edition of the present work, many physicians, not unworthy of notice, followed this old method, and treated the disease, from mere carelessness, as it was treated by the Spaniard Da Castro.

But, in addition to what has been stated, the acute observer and excellent practitioner, whose work we are considering, was soon led to perceive how little conformable to reason was the doctrine just described. This was indeed expressly declared by the great Sydenham, in a proposition adduced by the author, that this false idea of malignity had been more destructive to human nature than the invention of gunpowder; since it led many physicians to the use of cordials, alexipharmics, and a heating regimen, when they should, instead of them, have employed the most cooling remedies. But, unfortunately, Sydenham, for whom all show express veneration, or affect to do so, was not heard; nor were the opinions of other eminent authors, who expressed sentiments equally judicious, attended to on this occasion. The well-established facts were not borne in mind, of diseases of this species having been treated solely, from the first period to the last, and during the appearance of the most manifest nervous phenomena, merely by the external and internal use of iced-water, by Monardes, Todaro, and Cirillus; or, at least, admitting these facts, they endeavoured to maintain that ice, in such circumstances, acted by corroborating the system. The opinions of which we speak were generally diffused amongst physicians, according to which they admitted this dream of debility, when the work on the fever of Genoa, with novel observations so rigorously instituted, appeared to effect the overthrow of that ancient error.

And what can be opposed to this? Perhaps the veracity of the statements may be questioned? But the facts occurred in one of the first-rate cities in Italy, under the eyes of many physicians whose names are mentioned in the work, some of whom are yet living, as well as a greater proportion of the patients whose histories the author has related in it. They occurred under the observation of physicians, many of whom were followers of the contrary method, and were interested in defending their own conduct by accusing that of the author. After three editions of this work, no one has ventured to place in controversy the truth of those statements, although a legion of physicians have unchained themselves on other pretences against the founder of the contra-stimulant doctrine. And, indeed, should the controversy be again agitated, the last petechial epidemic which unhappily spread throughout Italy, has given but little occasion to physicians to contest the truth of what has been related.

It may perhaps be said, that the nature of that particular epidemic, as well as the more recent one, and the particular circumstances of the patients, did not present the predominance of malignity and debility; and that there were not witnessed, very fortunately, that complication with hypo-asthenia which, in other epidemics and other patients, a great number of ancient and modern practitioners may have observed. But, if this malignity, if this complication, has apparent and sensible characters; if these characters are not entirely metaphysical; and if they are said, by all ancient and recent observers, to be apparent by prostration of the pulse and strength, apparent vital languor, and the other signs that classic writers have extensively enumerated, not one of them was wanting in the greater proportion of those cases; and they will all be found there, even in the highest degree, joined, moreover, with preceding causes that are considered to be the most powerfully depressing; as the reader will perceive who has attended to the circumstances which have been premised.

Perhaps, too, it may be pretended, that, to the constancy of the fortunate success obtained by Professor G. Rasori, may be counterpoised the equal felicity of success, at other times, and by other physicians, certainly not favourers of these new sentiments, by using a method modified according to circumstances, by stimulating in the nervous period according to the ancient rules? Perhaps, too, some may thence consider that this compromises the fact, or favours the ancient, not less than the modern, opinion; or shows, at least, that the fundamental maxims, on which it rests as on a firm basis, are highly equivocal and fallacious? But those who use these arguments have not taken into consideration a great number of circumstances which concur to refute them.

In the first place, we will concede to a certain degree, that some of the supporters of the doctrine of malignity were really so fortunate in many of their cures modelled on those pathological principles; but did not the generality of their medical measures owe their favourable success to the equivocal circumstances of their having been administered as stimulants and corroborants? Let us examine impartially their clinical conduct. How did they act when the pretended nervous stage commenced?

They relinquished, it is true, the use of venesection and antimonials. They renounced, after the seventh day, the exhibition of tartarized and nitrated drink. They gave wine and ether, which certainly stimulate; musk and opium, which stimulate: but, at the same time, they did not forego leeches to the nostrils or to the hæmorrhoidal vessels, cups with scarification applied to the head, to withdraw the *peccant humour*, or remove irritation, or rouse the patient from stupor. Neither did they deny the use of ice, to *corrugate the too-relaxed fibres of the stomach;* they covered the abdomen with ice, to corrugate it, and to relieve meteorism; and they gave mineral lemonade, to suppress diarrhœa, or the vegetable acids, to *resist putridity*,

They at the same time ordered tepid, if not cold, baths, to eliminate from the skin the contagious miasmata. They also employed purgative enemas; produced blisters, and kept them open; recommended copious drinking of warm water as a diluent; prescribed kermes when the chest was affected, calomel, to *neutralize the contagion*, and atropa belladonna, to remove delirium. At the same time, whenever the pulse rose or became tense, on every attack of ferocious delirium, considering these as signs of irritation, or of phlogosis superadded to the nervous disorder, they recurred to venesection, or at least to bleeding with leeches and scarification; on every indication of new oppression, they purged, or gave an emetic; and we see, throughout the whole of their conduct in the treatment, the curious and contradictory alternative of distrusting to-day what they had done on the preceding one, and so throughout. They also conformed to the practical precepts of the classics, that the restoratives, corroborants, and excitants, should be given in proportion to the manner in which the patient would bear them, and at first very sparingly; and this parsimony was especially applied to opium, wine, ether, ammonia, and to the things termed heating; and that the remedies which may be exhibited freely are, contrayerva, arnica, serpentary, and similar substances: in short, those whose qualities, if not sedative, as we pretend, are at least subject to controversy. In most cases, too, the debilitating treatment was used whilst the disease maintained its severity, and the other was not resorted to until it was already conquered.

From these considerations, every one will perceive that the favourable results of a great number of cases treated in the preceding manner, opposed to those of a much greater number of cures effected by the use of debilitating measures from the commencement to the termination of the disease, prove nothing against the latter. That the arguments might have any degree of force, it would be necessary to show that such favourable results, in a considerable number of cases, had arisen from the treatment conducted by wine, ether, opium, and salt of hartshorn, from the beginning to the end of the malady; as, on the other hand, a multitude of cases were treated throughout with tartar-emetic, nitre, cremor tartar, bleeding, and leeches, alone. There being by no means any parity of circumstances, the whole advantage is on our side,—the whole disadvantage presses on the favourers of the mixed method. Whilst, then, they wait for further experience to remove this disadvantage, let us in the mean time consider as of but little importance the difficulty here opposed to us, in the fortunate success of the mixed method on some occasions, whilst, on others, it has been followed by different results.

But, we think, in the second place, and all those will agree with us who are acquainted with the recent history of medicine,—we think, that there is no occasion for waiting for the consequences of the experience just alluded to. We may anticipate their results. Setting out from the impossibility that a contradictory method might be beneficial where the opposite method is beneficial, we have an argument *a minori ad majus*, to use the language of the schools, that we may thus reveal the success that should be expected from such a trial. Since the mixed method succeeded so unfavourably in the former epidemic at Genoa, and in the more recent one, and modern and ancient practitioners have in all times had to repent having lavished stimulants even where they alternated remedies which subtracted from the influence of the measures which increased the excitement, we may previously know what will at best be the result of the treatment, purely and powerfully, of the stimulating kind.

The existence of malignity, then, in petechial fever, is a mere chimera; and this important fact, seen long since, in a glimmering light, by Sydenham, and then by Stoll, and by many others, has only lately been fully discerned by one of the founders of the reform in pathology: but, if the existence of that malignity be chimerical in such a species of disease, as the work of Rasori teaches us; and if the presence of low and small pulse, prostration of physical power, sensible depression of vitality, tremors, convulsions, and stupor, may co-exist with a state of excitement; what, then, becomes of this malignity in all analogous maladies? What, also, becomes of this pretended debility of Brown in such circumstances? What reason had he for believing in the existence of malignity in cases perfectly similar to the present, in confluent variola, or in the other exanthemata, in low nervous fever, as it is so improperly termed, in typhus or other febrile diseases, which, in early times, were so efficaciously treated by the antiphlogistic method, and the propriety of which the most modern have placed beyond dispute.

We have here only proposed what the preceding phenomena absolutely and rigorously substantiate; and we have elsewhere had frequent occasion to observe how facts, examined with severe criticism, lead us to think, that in no sudden disease have the phenomena any thing in common with real pathological debility,—we mean to say, idiopathic debility. We fortunately readily find, in other cases, characters sufficiently differing from those above mentioned, to enable us to avoid confounding them, in the signs of real debility or contra-stimulation. Let us, then, guard against exclaiming, with some, Debility does not exist, according to the modern doctrine: the old, and, as they were considered, true signs of it, fail to demonstrate it! Let us, however, endeavour not to confound mere appearances with reality, and finally to establish this first law, which results in the first instance from the work we have examined: *The false debility of the patients in the disease above described, is an illusion, a mere appearance; and the disease is successfully combatted solely by a method rigorously and purely antiphlogistic.*

---

### *The History of a remarkable Case of Thoracic Disease.*
### By Dr. Kohler, of Berlin.*

Paulina A—, six years and a half old, had enjoyed in her infancy and the early part of her childhood, a good state of health: she had experienced the ordinary disorders of children, but no other remarkable

* *Achter und Neunter Jahresbericht des Königl. Poliklinischen Instituts der Universität zu Berlin*, von den Jahren 1817 und 1818. Herausgegebenen von Prof. Hufeland. Journal der Practischen Heilkunde, Junius 1819.—A translation of malady. About six months since, she began to show evidences of disease: her appetite became impaired, she was disposed to solitude, and would often leave her childish amusements to go and weep alone, without any evident cause. The parents attributed this conduct chiefly to a peevish disposition, and thought moral and domestic management most proper for its correction. This state of disorder, however, soon assumed a more serious character: the child daily became enfeebled and emaciated, and a swelling appeared outwardly on the left side of the chest. The parents then brought her to the Royal Clinical Institution, where she came under my care on the 5th of November, 1817. The following were the circumstances developed on a careful examination of the history and present state of the disease.

On the left side, near to the sternum, about the region of the fourth, fifth, and sixth, ribs, there was a preternatural protuberance of the thoracic parietes: the skin over this was of a bluish colour, from congestion of blood in the veins. Powerful pressure on this part caused darting painful sensations. A throbbing of the heart was clearly evident externally; and, on the application of the hand to the swelling, the violent action of that organ became more forcibly distinguished. The pulse was quick, full, and regular; but there was a difference in respect to its strength in the two arms: it was fuller and harder in the left than in the right one. The cheeks of the patient were of a marked red colour; but, according to the father's statement, when she fell into fits of passion, and which she was much inclined to, they, as well as the lips, became of an extraordinary pallid hue. There was a remarkably bluish tinge throughout the scleroticæ of both eyes. The child complained of oppression of respiration, darting pains in the chest, especially in the region of the swelling, with which the cough she had, and the throbbing of the heart, were increased; she had also pains in the head, flushing of heat about her, and a disinclination for food. Her sleep was unquiet, and commonly disturbed by dreams, in which she often cried out aloud for help, waking her parents; but she could give no reason for this, and did not complain then of any severe pain. The evacuations from the bowels were natural in appearance, as well as the urinary secretion. With the foregoing circumstances there were signs of worms present,—that is, dilatation of the pupils, itching in the nostrils, occasionally attacks of pain in the belly, drawing-up of the feet during sleep, and occasional fits of voracious appetite for bread, and the like substances. The functions of the rest of the abdominal viscera appeared to be in the natural state. The general appearance of the habit presented signs of a scrofulous disposition. But the immediate cause of the present disease was not at all evident. The father recollected her having had a fall on the chest a few years since, but the child had received from it no apparent injury.

There were, however, existing evidences of an inflammatory dispo-

---

this history will only be here given; as one of the most important circumstances in the subject of it, the pervious state of the foramen ovale, will be considered in a general manner in the Historical Essay for the period just elapsed.—Edit.

sition that should be combatted: the pain, the cough, and the inordinate force of the actions of the vascular system, showed it in a decisive manner. But here was an important question to be determined,—whether the functions of the heart were disordered from actual organic lesion, of an inflammatory nature, of its own structure; or whether some other of the thoracic organs was the seat of the disease, and that which produced the external swelling?

The prognosis was necessarily unfavourable. The mode of cure was founded on the following indications: 1°, to combat the inflammatory state of the vascular system by sedatives, and by lessening the quantity of blood; 2°, to support the strength of the patient by restoratives not possessing heating qualities.

Four leeches were ordered to be applied to the chest, and the bleeding afterwards promoted for several hours; and a mixture of infusion of digitalis, with nitre, to be taken inwardly.

*Nov. 15th.*—The child is altogether better: she sleeps more quietly, and her appetite for food begins to improve. She is also somewhat livelier, and more inclined to partake of the ordinary amusements of children. The state of the pulse, the throbbing of the heart, and the dyspnœa, are still the same. The signs of the presence of worms in the intestines are much as before; the patient has complained of pain in the belly. I therefore prescribed, in addition to the former medicines, that a tea-spoonful of the anthelminthic electuary of the Prussian Pharmacopœia should be given every morning and evening. The swelling to be fomented with cold water.

*Nov. 20th.*—Several ascarides have been passed from the bowels. The inflammatory symptoms are not lessened. The pains in the chest, and the cough, are somewhat alleviated: but the child is more low-spirited; she has wept almost all the day, and her sleep has been very unquiet, and much disturbed by dreams. The state of the pulse and respiration is in the same state as before. Four leeches to be applied to the chest; the anthelminthic electuary and cold fomentation to be continued, and the quantity of digitalis in the mixture to be increased.

*Dec.* 2.—The mixture has produced sickness, vomiting, and head-ach. No more worms have been passed. The throbbing of the heart is not quite so violent: in other respects, the state of the patient is similar to that of the 20th of November. The dose of the digitalis to be diminished; and a mixture made with infusion of valerian, laurel-water, and extract of hyosciamus, to be taken occasionally. The use of the electuary, and the fomentation, to be persisted in; but the water to be rendered colder by admixture with ice or snow.

*Dec.* 20.—Some more ascarides have been passed. The pulse is not quite so full, and less frequent; the appetite is not quite so good as it was a few days since; the sleep has been much disturbed by dreams; the signs of worms are still very evident; the pains in the chest occur less frequently, and are also less severe. The infusion of digitalis with nitre to be continued; a decoction of staves-acre, with extract of wormwood and tartrate of potash, to be also administered; the mixture of valerian, &c. and the application of cold water externally, to be continued.

*Jan.* 2, 1818.—The medicines have produced vomiting; no more worms have been passed; the swelling becomes more remarkable about the upper part of the sternum, in which part the greatest pain is now experienced. The infusion of valerian and laurel-water, and the other medicines, to be continued; and a glyster of milk, with garlic boiled in it, to be administered daily.

*Jan.* 10*th*.—The patient is beginning to be more lively; her sleep is quieter; the pain is less severe; and the vomiting has ceased. The glyster, appearing to be beneficial, to be repeated.

*Jan.* 18*th*.—Pulsation of the carotid arteries is remarkably evident. The child tosses herself about, and talks much, whilst asleep; the mother remarks that she has become much emaciated; the pulse is full and frequent. Three leeches to be applied to the chest; and, as the digitalis has produced narcotic effects, it is to be discontinued; the other measures to be persevered in.

The character of the symptoms of the thoracic disease varied but little until the 2d of February. The febrile excitement is now less considerable; a scrofulous enlargement of the axillary glands has appeared. There is now an accessory catarrhal cough: the cold fomentation, therefore, to be discontinued. The upper part of the abdomen is hard and distended; the pains in the belly are violent, and of longer duration than heretofore. There is more evident swelling about the subclavicular region. The antiphlogistic and anthelminthic measures had been employed to this time; I now determined to change them for some light tonic medicines, and ordered the following: ℞. *Rad. bardan.* ʒ vi. *coque in aq. font. lib. i ad* ℥ vi. *col. adde liq. terr. foliat. tartar.* (*potassæ acetas*) ʒ iii. *ext. taraxac. ana unc. semisse: syr. de althæa,* ℥ i. M. A small table-spoonful to be taken every second hour.—℞. *quoque, Æthiop. antimon.* gr. vi. *pulv. digitalis purp. gr. semisse; sapon. venet. exsiccat.; sacch. albi, ana* gr. x. *M. f. pulvis:* half of which to be taken every morning and evening. Besides the above medicines, I ordered a glyster made with decoction of taraxicum and succory, with the addition of a little soap, to be given daily.

*Feb.* 16*th*.—The stomach would not bear the powders; they are therefore to be discontinued. The appearance of the countenance of the child is somewhat better; she is more active and cheerful. The mixture ordered the 2d of February to be continued; and the abdomen to be rubbed with a volatile camphorated liniment.

*Feb.* 22*d*.—She is evidently and surprisingly better. The same measures to be continued.

*Feb.* 28*th*.—Symptoms of dropsy have now first made their appearance. There is œdema of the feet, face, and eye-lids; the appetite for food is almost totally lost; the urine has become small in quantity, and it is voided with pain. The child sleeps almost throughout the day, and is delirious when asleep; the throbbing of the heart is less violent. The prognosis is of the most unfavourable kind: death appears inevitable. However, in order to give the patient some relief, and to increase the vital powers in general, I ordered a mixture to be taken composed of *inf. rad. Levistici* and *valerianæ*, with *ext. Chinæ* and *sp. æth nitrici;* the abdomen to be embrocated with petroleum; the

œdematous swelling of the feet to be rubbed with flannel several times during the day.

The child took but little of the medicines. Her state is hourly becoming worse; she has intense thirst; the febrile excitement is more violent, and debility increases daily.

*March 3d.*—A colliquative diarrhœa contributes to exhaust the patient's strength; the urine is evacuated unconsciously and involuntarily. It only remains to render her a little relief by mild opiates, and other obvious similar means. She died calmly and easily on the 10th of March, at noon, without any previous convulsive actions of the respiratory organs or any great agony, wholly retaining her intellects, and the power of speech, to the last moment of her existence.

Dissection of the body was made on the 11th of March, and furnished the following results: The appearance of the corpse was "cachectic;" the upper part of the body was very much emaciated; the feet tumid from œdema. The cartilaginous extremities of the first four ribs, on both sides, about their union with the sternum, were wholly changed into a caseous, pulpy, mass; the sternum itself, on the inner surface of its upper part, was affected with caries. The lungs were externally of a dull whitish-yellow colour; but they were not, in their more internal structure, much altered by disease: the left lung, however, adhered to the diaphragm and the intercostal muscles. On opening the pericardium, it was found to contain about half an ounce of a turbid, yellowish-coloured, serum; and, on the lower part of that membrane, there was a small cartilaginous concretion,* the form of which cannot be better described than by stating it to be similar to a large os sesamoideum. The heart itself was in a loose, flaccid, state: the substance of its right ventricle was, in relation to that of the left ventricle, unusually thin. The foramen ovale was so far open, that a common quill might be easily passed through it. The valves of the heart, with those of the great vessels, as well as the latter themselves, were in the natural state. The right auricle contained some blood; the rest of the cavities were devoid of that fluid. The liver, as is commonly the case in children, extended into both hypochondres, and on its surface a considerable number of lymphatic vessels were very evident: the interior structure of this viscus presented nothing remarkable. The stomach was nearly full of the fluids that had been taken as drink; the pyloric aperture was in a morbid state, as well as the duodenum, which felt thicker than natural. The small intestines were empty; but their vessels were fully injected with blood, and they presented two small spots of ulceration, which however had only destroyed the external coat, and produced a little alteration of the appearance of the others. The large intestines were free from fæces; their vessels, in some places, were injected with blood. The mesenteric glands were generally very much enlarged: some of them were of the size of a hen's egg; and these, on being cut into, displayed a

* The precise part of the pericardium on which this was situate, is not mentioned by Dr. Köhler; neither is it clearly stated in what way it was attached to that membrane. The original is,—*Am untern Theil des Pericardium sah ich einige kleine, knorpelichte concremente.*—Edit.

white caseous mass. The spleen and kidneys were in the healthy state. The bladder was contracted into a small compass in the lower part of the pelvis, and contained a little urine; its structure was somewhat thickened. The opening of the head was not permitted.

### Medical and Philosophical Intelligence.

